# Post hoc analysis of the glutamics–trial: intravenous glutamate infusion and use of inotropic drugs after cabg

**DOI:** 10.1186/s12871-016-0216-z

**Published:** 2016-08-02

**Authors:** Mårten Vidlund, Bashir Tajik, Erik Håkanson, Örjan Friberg, Jonas Holm, Farkas Vanky, Rolf Svedjeholm

**Affiliations:** 1 Department of Cardiothoracic Surgery and Cardiothoracic Anaesthesia, Faculty of Medicine and Health Sciences, Division of Cardiovascular Medicine, Linköping University, Linköping, Sweden; 2 Department of Cardiothoracic and Vascular Surgery, Faculty of Medicine and Health, Örebro University, Örebro, Sweden

**Keywords:** Coronary artery bypass surgery, Intensive care, Postoperative complications, Inotropic drugs, Milrinone, Epinephrine, Glutamate

## Abstract

**Background:**

Intravenous glutamate reduced the risk of developing severe circulatory failure after isolated coronary artery bypass graft surgery (CABG) for acute coronary syndrome (ACS) in a double-blind randomised clinical trial (GLUTAMICS-ClinicalTrials.gov Identifier:NCT00489827). Here our aim was to study if glutamate was associated with reduced the use of inotropes.

**Methods:**

Post-hoc analysis of 824 patients undergoing isolated CABG for ACS in the GLUTAMICS-trial. ICU-records were retrospectively scrutinised including hourly registration of inotropic drug infusion, dosage and total duration during the operation and postoperatively.

**Results:**

ICU-records were found for 171 out of 177 patients who received inotropes perioperatively. Only one fourth of the patients treated with inotropes fulfilled study criteria for postoperative heart failure at weaning from cardiopulmonary bypass (CPB) or later in the ICU. Inotropes were mainly given preemptively to facilitate weaning from CPB or to treat postoperative circulatory instability (bleeding, hypovolaemia). Except for a significantly lower use of epinephrine there were only trends towards lower need of other inotropes overall in the glutamate group. In patients treated with inotropes (glutamate *n* = 17; placebo *n* = 13) who fulfilled study criteria for left ventricular failure at weaning from CPB the average duration of inotropic treatment (34 ± 20 v 80 ± 77 h; *p* = 0.014) and the number of inotropes used (1.35 ± 0.6 v 1.85 ± 0.7; *p* = 0.047) were lower in the glutamate group.

**Conclusions:**

Intravenous glutamate was associated with a minor influence on inotrope use overall in patients undergoing CABG for ACS whereas a considerable and significant reduction was observed in patients with heart failure at weaning from CPB.

**Electronic supplementary material:**

The online version of this article (doi:10.1186/s12871-016-0216-z) contains supplementary material, which is available to authorized users.

## Background

The GLUTAMICS-trial investigated if intravenous glutamate infusion given in association with surgery for acute coronary syndrome could prevent myocardial injury, postoperative heart failure and reduce mortality [1]. The study was negative with regard to the primary endpoint, which was a composite of postoperative mortality, perioperative myocardial infarction and left ventricular failure at weaning from cardiopulmonary bypass (CPB). However, intravenous glutamate reduced the relative risk of developing severe circulatory failure by more than 50 % in most high-risk groups. Patients with heart failure at weaning from CPB required markedly shorter ventilator treatment and ICU-stay if they were treated with glutamate. These results are compatible with a beneficial effect of glutamate on post-ischaemic myocardial recovery after coronary artery bypass surgery (CABG).

However, it can be argued that other factors such as differences in the use of inotropic drugs could have explained the results. If glutamate enhances myocardial recovery in post-ischaemic heart failure one would expect it to be accompanied by a reduced need for inotropic drugs. The primary aim of this study was, therefore, to investigate if intravenous glutamate infusion influenced the use of inotropic drugs in patients operated for acute coronary syndrome in the GLUTAMICS-trial. The secondary aim was to investigate if glutamate due to its suggested vasodilator properties influenced the need for vasoconstrictor therapy (noradrenaline) early postoperatively.

## Methods

### Patients

This study is a post hoc analysis of a double-blind prospective randomised clinical trial, the GLUTAMICS-trial [1]. Inclusion criteria were CABG for acute coronary syndrome. Patients were eligible for inclusion regardless if the procedure was done on-pump or off-pump.

Exclusion criteria were: informed consent not possible because of critical condition or other reason, age > 85 years, body weight >125 kg; food allergy known to have caused rash, flush or asthma; preoperative use of inotropic drugs or mechanical circulatory assist, preoperative dialysis, redo-procedure, unexpected intraoperative finding or event that increased the magnitude of the procedure to overshadow the originally planned procedure.

**Fig. 1:**
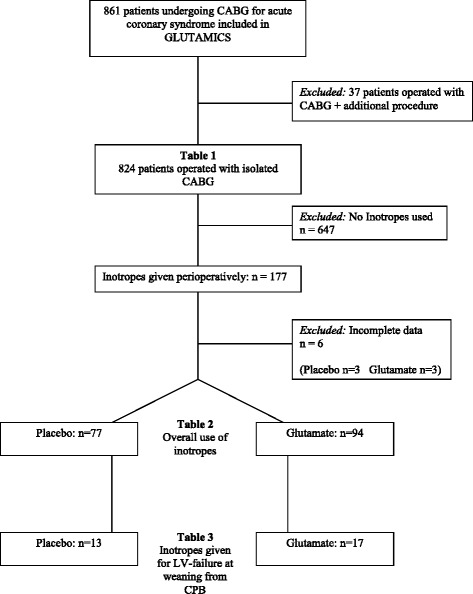
Flowchart of patient selection and presentation of results at three different levels

The original trial was planned for 2214 patients but the trial was terminated after 861 patients as prespecified stopping criteria per protocol were fulfilled at interim analysis as previously reported [1]. For this particular analysis a small heterogeneous group of patients having additional procedure to CABG was excluded (Fig. 1). Eight hundred twenty four patients in the GLUTAMICS-trial were operated with isolated CABG for acute coronary syndrome between October 4, 2005 and November 12, 2009 at three Swedish Cardiac Surgery Centres (University Hospital of Linköping, Örebro University Hospital and Blekinge County Hospital in Karlskrona).

Patients were randomised to blinded intravenous infusion of 0.125 M glutamate or saline solution at a rate of 1.65 ml/kg body weight and hour started at the induction of anaesthesia and stopped 2.5 h after declamping the aorta or when total of 500 ml had been infused. Further details on the glutamate solution and the GLUTAMICS-trial are given in the Additional file 1.

Patients receiving inotropes were identified by the aid of the Case Report Form (CRF), the institutional database and ICU-records. Use of inotropes was registered in the CRF at weaning from CPB and on admission to ICU. Furthermore, use of inotropes exceeding 30 min was registered by the attending physicians in the institutional database. Finally the ICU records of all patients staying longer than 24 h were retrospectively investigated to identify use of inotropes that had not been recorded in the CRF or database. Complete ICU-records were found for 171 out of 177 patients who received inotropes during this period (Fig. 1). These medical records were then scrutinised by an investigator (BT) previously not involved in the GLUTAMICS-trial. Data retrieval included hourly registration of inotropic drug infusion, dosage and total duration intraoperatively and postoperatively.

Inotropic drugs used during the study period were adrenaline, milrinone (Corotrop™), levosimendan (Simdax™) and dopamine. Adrenaline and milrinone were the most used drugs whereas levosimendan and dopamine only were used in a small fraction. Therefore we present the results for inotrope use overall and separately only for adrenaline and milrinone treatment.

Noradrenaline was used liberally as a vasoconstrictor and its dosages were registered for the first 15 postoperative hours.

### Definitions

Use of inotropes was defined as any use of inotropic drugs (except noradrenaline) regardless of indication, duration or dosage. Participating centres and individual clinicians were free to institute inotropic drugs according to their choice and usual practice.

Preemptive use of inotropic drugs to facilitate weaning from CPB was defined as use of inotropic drugs before weaning from CPB in patients that according to prespecified criteria were deemed, by the blinded clinical endpoints committee, not to have had heart failure at weaning. If, however, the patient fulfilled prespecified criteria for heart failure at weaning use of inotropes was considered “treatment” regardless if the drugs had been instituted before weaning or instituted because of weaning problems.

Prespecified criteria were used by a blinded endpoints committee to determine if heart failure was present. Briefly clinical and echocardiographic signs of heart failure as a cause of low mixed venous oxygen saturation (SvO_2_) were used to diagnose postoperative heart failure [2–4]. A surgical pulmonary artery catheter was introduced in all patients for sampling of SvO_2_ [2, 3]. SvO_2_ was measured in every patient at weaning from CPB, 5 min after protamine administration and on admission to ICU and whenever clinical condition of the patients required evaluation of the haemodynamic status. If the rare cases when a patient required more meticulous monitoring to guide haemodynamic treatment a Swan-Ganz catheter was employed. Detailed criteria are given in the Additional file 1.

Postoperative heart failure was categorised into heart failure evident at weaning from cardiopulmonary bypass or late circulatory failure presenting after apparently uncomplicated weaning. Circulatory failure was classified as late if it became evident after weaning from CPB and it was categorised as cardiac or non-cardiac in origin.

Severe circulatory failure was defined as heart failure leading to death or requiring ICU stay ≥ 48 h with intra-aortic balloon pump for ≥24 h or inotropic agents in dosages according to Additional file 1 for ≥24 h.

Postoperative mortality was defined as death within 30 days of surgery. Hospital mortality was defined as death during the first hospitalisation period including stay at the referral hospital after discharge postoperatively. Cardiac cause of death was assessed by the endpoints committee.

### Statistics

Two-sided Fisher’s exact test was used was used for comparison of dichotomous variables. Two-sided Students *t*-test or Mann-Whitney *U* test as appropriate were used for comparison of continuous variables. Statistical significance was defined as *p* < 0.05. The data are given as percentages or means ± standard deviation.

## Results

### Preoperative data

Preoperative data for patients undergoing isolated CABG for acute coronary syndrome in the GLUTAMICS-trial is given in Table 1. Despite randomisation there were minor differences with significantly more patients having left main stenosis and extra-cardiac arterial disease in the glutamate group. There was also a trend toward a higher risk profile according to EuroSCORE in the glutamate group.

**Table 1:** Preoperative, intraoperative and postoperative data in patients undergoing isolated CABG for acute coronary syndrome in the GLUTAMICS-trial

	Placebo *n* = 413	Glutamate *n* = 411	*p-value*
Preoperative data			
Age (years)	68 ± 9	68 ± 9	0.30
Female	18.9 %	17.5 %	0.65
Weight (kg)	83 ± 15	81 ± 14	0.25
Length (cm)	173 ± 8	173 ± 8	0.59
BMI (kg/m2)	27 ± 4	27 ± 4	0.10
B-Hb (g/l)	137 ± 14	137 ± 14	0.68
p-Creatinine (μmol/l)	97 ± 29	98 ± 27	0.60
Hypertension	60 %	56 %	0.29
COPD	5.1 %	7.9 %	0.09
Diabetes	26 %	24 %	0.50
Extra-cardiac arterial disease	9 %	14 %	*0.049*
CCS class IV angina	56 %	55 %	0.73
Left main stem stenosis	34 %	42 %	*0.02*
Myocardial Infarct < 24 h	1.2 %	0.2 %	0.22
Moderate-Severe LV-dysfunction	18.6 %	18.0 %	0.86
EuroSCORE	4.9 ± 2.7	5.2 ± 2.7	0.10
Intraoperative data			
Number of bypasses	3.9 ± 1.1	4.1 ± 1.0	0.18
Cross-clamp time (min)	52 ± 18	51 ± 18	0.51
CPB time (min)	80 ± 25	81 ± 28	0.61
Indications for inotropes			
Preemptively before weaning from CPB	9.0 %	9.7 %	0.81
LV-failure at weaning from CPB	3.4 %	4.4 %	0.48
Late onset cardiac failure	1.9 %	1.9 %	1.0
Cardiac failure postoperatively (total)	5.3 %	6.3 %	0.54
Late circulatory failure non-cardiac	3.4 %	1.5 %	0.11
Other	0.9 %	5.4 %	*0.001*
Circulatory treatment			
Inotropes (total)	18.6 %	22.9 %	0.15
Adrenaline	11.9 %	16.1 %	0.09
Milrinone	9.4 %	9.0 %	0.90
Levosimendane	3.6 %	3.4 %	1.0
Dopamine	1.7 %	2.7 %	0.35
Intra-aortic balloon pump	1.2 %	0 %	0.06
Postoperative outcome			
Time on ventilator (h)	13 ± 54	10 ± 37	0.35
ICU stay (h)	31 ± 58	30 ± 52	0.77
Severe circulatory failure	3.9 %	1.5 %	*0.049*
Stroke within 24 (h)	1.5 %	1.0 %	0.53
Cardiac mortality	0.7 %	0.2 %	0.62
30 days mortality	1.0 %	0.7 %	0.71
Hospital mortality	1.2 %	1.2 %	0.74
Postoperative outcome			
Time on ventilator (h)	13 ± 54	10 ± 37	0.35
ICU stay (h)	31 ± 58	30 ± 52	0.77
Severe circulatory failure	3.9 %	1.5 %	*0.049*
Stroke within 24 (h)	1.5 %	1.0 %	0.53

*BMI* body mass index, *COPD* chronic obstructive pulmonary disease, *LV* left ventricular, *CPB* cardiopulmonary bypass, *ICU*, intensive care unit

### Intraoperative data

Intraoperative data for patients undergoing isolated CABG for acute coronary syndrome in the GLUTAMICS-trial is given in Table 1. No significant differences between the groups were observed.

### Postoperative data

Postoperative data for patients undergoing isolated CABG for acute coronary syndrome in the GLUTAMICS-trial is given in Table 1. The incidence of severe circulatory failure was significantly lower in the glutamate group (1.5 % v 3.9 %; *p* = 0.049).

Postoperative data for all patients treated with inotropes after isolated CABG for acute coronary syndrome in the GLUTAMICS-trial is given in Table 2. The incidences of severe circulatory failure (6.2 % v 17.5 %; *p* = 0.03) and the need for intra-aortic balloon pump (0 % v 5.2 %; *p* = 0.04) were significantly lower in the glutamate group.

**Table 2:** Circulatory treatment and postoperative outcome in all patients treated with inotropes

	Placebo *n* = 77	Glutamate *n* = 94	*p-value*
Circulatory treatment			
Inotropes (total)	100 %	100 %	
Inotropes duration (hours)	38 ± 46	29 ± 30	0.14
Number of inotropes	1.4 ± 0.6	1.3 ± 0.6	0.45
Adrenaline	63.6 %	70.2 %	0.41
Adrenaline duration (hours)^a^	28 ± 49	17 ± 25	0.049
Adrenaline duration (hours)^b^	44 ± 55	24 ± 27	0.01
Average max dosage μg/kg/min^b^	0.028 ± 0.016	0.028 ± 0.020	0.94
Milrinone	50.6 %	39.4 %	0.16
Milrinone duration (hours)^a^	14 ± 25	9 ± 19	0.14
Milrinone duration (hours)^b^	27 ± 30	22 ± 25	0.44
Average max dosage μg/kg/min^b^	0.40 ± 0.14	0.36 ± 0.12	0.28
Noradrenaline	71.4 %	70.2 %	1.0
Average max dosage μg/kg/min^a^	0.084 ± 0.093	0.068 ± 0.089	0.24
Average max dosage μg/kg/min^b^	0.118 ± 0.090	0.097 ± 0.092	0.20
IABP	5.2 %	0 %	*0.04*
Postoperative outcome			
Time on ventilator (hours)	34 ± 95	26 ± 74	0.54
ICU stay (hours)	63 ± 98	59 ± 78	0.79
Severe circulatory failure	17.5 %	6.2 %	*0.03*
Stroke within 24 h	2.6 %	3.2 %	1.0
Cardiac mortality	2.6 %	1.1 %	0.59
30 day mortality	1.3 %	2.1 %	1.0
Hospital mortality	2.6 %	3.2 %	1.0

*IABP* intra-aortic balloon pump, *ICU* intensive care unit

^a^average for all patients treated with inotropes

^b^average for patients treated with specific drug

Postoperative data for patients treated with inotropes because of heart failure at weaning from CPB after isolated CABG for acute coronary syndrome in the GLUTAMICS-trial is given in Table 3. Glutamate treated patients had a significantly lower incidence of severe circulatory failure (11.8 % v 61.5 %; *p* = 0.007), shorter ventilator treatment and shorter stay in the ICU.

**Table 3:** Circulatory treatment and postoperative outcome in patients treated with inotropes because of heart failure on weaning from CPB

	Placebo *n* = 13	Glutamate *n* = 17	*p-value*
Inotropes (total)	100 %	100 %	1.0
Inotropic duration (hours)^a^	85 ± 77	34 ± 20	*0.014*
Number of inotropes	1.8 ± 0.7	1.3 ± 0.6	*0.04*
Adrenaline	85 %	88 %	1.0
Adrenalin duration (hours)^a^	78 ± 82	25 ± 19	*0.016*
Adrenalin duration (hours)^b^	92 ± 82	28 ± 17	*0.007*
Average max dosage μg/kg/min^b^	0.031 ± 0.012	0.029 ± 0.030	0.73
Milrinone	54 %	35 %	0.46
Milrinone duration (hours)^a^	33 ± 52	9 ± 14	0.09
Milrinone duration (hours)^b^	60 ± 60	27 ± 8	0.20
Average max dosage μg/kg/min^b^	0.38 ± 0.15	0.36 ± 0.02	0.78
Noradrenaline	85 %	59 %	0.23
Average max dosage μg/kg/min^a^	0.089 ± 0.096	0.034 ± 0.033	*0.03*
Average max dosage μg/kg/min^b^	0.106 ± 0.095	0.058 ± 0.022	0.14
IABP	31 %	0 %	*0.03*
Postoperative outcome			
Time on ventilator (hours)	109 ± 192	6 ± 3	*0.033*
Time on ventilator >48 h	46 %	0.0 %	*0.003*
ICU stay (hours)	150 ± 186	45 ± 38	*0.031*
Severe circulatory failure	61.5 %	11.8 %	*0.007*
Stroke within 24 h	0.0 %	0.0 %	1.0
Cardiac mortality	15.4 %	5.9 %	0.56
30 day mortality	15.4 %	5.9 %	0.56
Hospital mortality	15.4 %	5.9 %	0.56

*IABP* intra-aortic balloon pump, *ICU* intensive care unit

^a^average for all patients treated with inotropes

^b^average for patients treated with specific drug

### Inotrope use

ICU-records were found for 171 out of 177 patients who received inotropes perioperatively. Details of inotrope use are given in Tables 1, 2 and 3. Only one fourth of patients treated with inotropes fulfilled study criteria for postoperative heart failure at weaning from CPB or later in the ICU. The other main indications to give inotropes were preemptive administration to facilitate weaning from CPB or to treat postoperative circulatory instability (bleeding, hypovolaemia). Except for a significantly lower use of adrenaline there were only trends towards lower need of other inotropes overall in the glutamate group (Tables 1 and 2).

In patients who fulfilled criteria for left ventricular failure at weaning from CPB (glutamate *n* = 17; placebo *n* = 13) the average duration of inotropic treatment (34 ± 20 v 80 ± 77 h; *p* = 0.014) and the number of inotropes administered (1.35 ± 0.6 v 1.85 ± 0.7; *p* = 0.04) were lower in the glutamate group (Table 3).

### Vasoconstrictor use

Details of noradrenaline use during the first 15 postoperative hours in patients treated with inotropes are given in Tables 2 and 3. In patients treated with inotropes 71 % in the control group and 70 % in the glutamate group also received noradrenaline. The maximum dosage given did not differ significantly between the groups. Noradrenaline requirements were lower in the glutamate group among patients with heart failure at weaning from CPB (Table 3).

## Discussion

This post-hoc analysis of the GLUTAMICS-trial demonstrates that glutamate treatment was associated with a reduced use of inotropes in patients with heart failure at weaning from CPB. Glutamate infusion was not associated with an increased need for vasoconstrictors suggesting that any vasodilator properties of glutamate in the dosage used is limited.

Postoperative heart failure or low cardiac output syndrome is the major cause for morbidity and mortality in cardiac surgery [5–8]. Already in the beginning of heart surgery it was noted that early death after cardiac surgery was related to low cardiac output postoperatively [9]. Although the hazards of low cardiac output syndrome have been known for a long time, there is no consensus on how to define postoperative heart failure. Furthermore, there are no generally accepted guidelines for treatment of postoperative heart failure [10–12]. Different strategies for treatment with inotropic, metabolic and mechanical support depend on local clinical practice or are up to the discretion of the individual physician [12].

Inotropic drugs improve cardiac output but this is achieved at the price of a marked increase in myocardial workload and oxygen consumption [13]. In the early postoperative period after cardiac surgery the heart is in a vulnerable state recovering from ischaemia [14, 15]. Inotropic agents can aggravate the consequences of ischaemia [16]. It has been shown that ischaemia and evolving myocardial infarction account for a large proportion of postoperative heart failure after CABG [6, 17].

The role of inotropes in cardiac surgery is therefore controversial [12]. Some authors claim that liberal use of inotropes and goal-directed haemodynamic therapy can improve outcome whereas other have reported that liberal use of inotropes was associated with increased morbidity and mortality [12, 18–20].

In clinical practice the use of inotropes in cardiac surgery varies much between different institutions and different physicians as evidence-based guidelines are lacking. In the present study only one fourth of the patients treated with inotropes fulfilled study criteria for heart failure.

Several biochemical mechanisms by which glutamate could influence outcome after myocardial ischaemia have been described [21–24]. Glutamate could enhance myocardial tolerance to ischemia by its role in the malate-aspartate shuttle transporting reducing equivalents across the mitochondrial membrane, thus facilitating anaerobic glycolysis by regulating the NAD/NADH balance in the cytosol. Glutamate also contributes to an alternative anaerobic pathway for regeneration of high-energy phosphates in the mitochondria by substrate level phosphorylation. Glutamate improves clearance of lactate and NH3 by taking part in reactions involving transamination of pyruvate to alanine and glutamate to glutamine. Glutamate could stimulate post-ischaemic recovery of myocardial oxidative metabolism and function due to its anaplerotic role; glutamate plays a key role for replenishment of Krebs cycle intermediates lost during ischaemia [21–24].

In animal in vitro and in vivo models numerous studies have shown that glutamate protects the myocardium from ischaemia and promotes recovery of oxidative metabolism after ischaemia [21, 25–27]. In humans intravenous glutamate infusion has been shown to promote metabolic and haemodynamic recovery after cardiac surgery [28, 29].

Promoting metabolic and functional recovery with metabolic support represents a novel concept in the treatment of heart failure after acute ischaemia. Intravenous glutamate improved metabolic and haemodynamic recovery early after CABG [28, 29]. Early clinical experience with intravenous metabolic support showed that the need for inotropes could almost be abolished while clinical outcomes with regard to postoperative mortality, postoperative renal dysfunction and long-term survival compared favourably with literature [4]. This encouraging experience contributed to the initiation of the GLUTAMICS-trial.

The GLUTAMICS-trial was negative with regard to the primary endpoint [1]. However, the study included a high proportion of low risk patients and furthermore the design of the primary endpoint suffered from liberal preemptive use of inotropes in patients anticipated to have weaning problems. It became evident at clinical endpoint committee meetings that preemptive use of inotropes prevented detection of weaning problems in patients who later developed severe circulatory failure. The secondary endpoint severe circulatory failure discriminated mild short-lasting heart failure at weaning from cardiopulmonary bypass from clinically significant heart failure requiring substantial circulatory support and leading to prolonged ICU stay or death. In the glutamate treated patients the relative risk of developing severe circulatory failure was reduced by more than 50 % in most risk groups undergoing isolated CABG [1]. For those who believe that inotropes are beneficial after cardiac surgery the present post-hoc analysis clearly shows that these results were not explained by more liberal use of inotropes in the glutamate group. On the contrary, glutamate infusion was associated with a reduced need for inotropes in patients fulfilling criteria for heart failure on weaning from CPB, which is compatible with the suggested mechanism of glutamate, i.e. to promote post-ischaemic recovery of the heart. These results also agree with previous clinical experience, which has showed that metabolic support can achieve excellent clinical outcomes with reduced need for inotropes [4]. The fact that glutamate had a minor impact on inotrope use overall is not unexpected given that only about one fourth of the patients treated fulfilled criteria for heart failure.

This post-hoc analysis is unique as it, to our knowledge, is the first to provide a detailed investigation on the hourly use of inotropes and vasoconstrictors postoperatively after cardiac surgery. The major limitation is the nature of the study. Post-hoc analyses should always be interpreted cautiously. The criteria used for postoperative heart failure in the GLUTAMICS-trial are inevitably debatable. Although this condition is a major cause for postoperative mortality generally accepted criteria for this diagnosis are lacking. To address this issue the investigators had based the criteria on variables documented with regard to outcome and provided the blinded clinical endpoints committee with strict prespecified criteria to minimise bias from individual clinical judgement [1–3].

A potential hazard of glutamate is that it acts as an excitotoxin, which could contribute to neurological damage under certain conditions. However, no increase in clinically evident neurological injury has been observed when glutamate enhanced cardioplegic solutions or intravenous infusions have been used in clinical practice [30, 31]. We could not detect any evidence for subclinical neurological injury associated with intravenous glutamate in the S-100B substudy of the GLUTAMICS-trial [32]. Another concern has been suggested vasodilator properties occasionally observed in association with glutamate enriched blood cardioplegia. In this post-hoc analysis no increase in the need for vasoconstrictors were observed with the dosage of glutamate used during first 15 postoperative hours when the effect of glutamate should have subsided. With the infusion rate used a decrease to normal blood levels of glutamate has been observed within 30 min after discontinuation of the infusion [33]. Thus, it appears that intravenous glutamate infusions in the dosages employed can be safely administered to patients undergoing cardiac surgery.

## Conclusions

Intravenous glutamate was associated a minor effect on inotrope use overall in patients undergoing CABG for acute coronary syndrome whereas a considerable and significant reduction was observed in patients with heart failure at weaning from CPB. Only about one fourth of the patients treated with inotropes in the GLUTAMICS-trial fulfilled criteria for heart failure.

## Abbreviations

ACS, acute coronary syndrome; CABG, coronary artery bypass graft surgery; CPB, cardiopulmonary bypass; CRF, case report form; GLUTAMICS, glutamate for metabolic intervention in coronary surgery; ICU, intensive care unit; NAD, nicotinamide adenine dinucleotide (oxidised form); NADH, nicotinamide adenine dinucleotide (reduced form)

## Additional file

Additional file 1: Details of intervention, study design, inclusion criteria, exclusion criteria, definition of endpoints, stopping criteria, data collection, routines for unblinding, clinical management and clinical endpoints committee. (DOC 72 kb)
