# Supplementary material for: Post hoc analysis of the glutamics–trial: intravenous glutamate infusion and use of inotropic drugs after cabg
Source: BMC Anesthesiol. 2016 Aug 2;16:54. doi: 10.1186/s12871-016-0216-z (PMC4971701; doi:10.1186/s12871-016-0216-z)
Supplement: Additional file 1: — Details of intervention, study design, inclusion criteria, exclusion criteria, definition of endpoints, stopping criteria, data collection, routines for unblinding, clinical management and clinical endpoints committee. (DOC 72 kb) [file 12871_2016_216_MOESM1_ESM.doc]

**Additional file 1**

# GLUTAMICS

# GLUTAmate for Metabolic Intervention in Coronary Surgery (ClinicalTrials.gov Identifier: [**NCT00489827**](http://clinicaltrials.gov/ct2/show/NCT00489827)).

The study was approved by the Swedish Medical Products Agency (151:2003/70403) and the Regional Ethical Review Board in Linköping (M76-05). Amendments were accepted by the Swedish Medical Products Agency 2006-08-31, 2007-05-08, 2007-11-01 and 2007-11-19.

External randomization in variable block sizes was done by Apoteket AB, Produktion & Laboratorier (APL), Box 6124, SE 90604, Umeå, Sweden.

External monitoring of all key data and unblinding procedures was done by an independent professional monitoring team (Clinical Research Support: http://www.orebroll.se/crs).

Recording of adverse events was done according to Good Clinical Practice standard.

**INTERVENTION**

**Glutamate solution**

500 ml 0.125 M solution of L-glutamic acid with pH 6.0 and 280 mosmol/kg containing L-glutamic acid 9.2 g, NaCl 0.8g, H_2_O ad 500 ml and NaOH quantum satis.

Production of glutamate solution and quality control was done by Apoteket AB, Produktion & Laboratorier (APL), Box 6124, SE 90604 Umeå, Sweden.

**INCLUSION CRITERIA**

Inclusion criteria were coronary artery bypass surgery for acute coronary syndrome. Patients were eligible for inclusion regardless if the procedure was done on-pump or off-pump or if the patient had a simultaneous valve procedure.

**EXCLUSION CRITERIA**

Exclusion criteria were, informed consent not possible because of critical condition or other reason, preoperative use of inotropic drugs or mechanical circulatory assist, preoperative dialysis, redo-procedure, unexpected intraoperative finding or event that increased the magnitude of the procedure to overshadow the originally planned operation, age > 85 years, body weight > 125 kg and food allergy known to have caused flush, rash or asthma.

**STOPPING CRITERIA**

Safety aspects had priority as stopping criteria. The following stopping criteria were prespecified.

The study would be terminated:

- If monitoring or interim analysis revealed an increased rate of stroke or mortality the study would be terminated before statistical significance was reached.

- If unexpected serious adverse events were detected in the glutamate group

- If clinical benefit regarding mortality (p<0.05) was reached for glutamate infusion at interim analysis

- If there was no statistically significant difference regarding markers of myocardial injury or renal function in favour of glutamate infusion at interim analysis

- If there was no absolute difference in the primary endpoint in favour of glutamate infusion at interim analysis

**PRESPECIFIED ENDPOINTS**

**PRIMARY ENDPOINT**

The primary endpoint was a composite of postoperative mortality (≤30 days), perioperative myocardial infarction* and left ventricular heart failure in association with weaning from cardiopulmonary bypass or after completion of off-pump coronary artery bypass surgery**.

***Perioperative Myocardial Infarct**

The criteria were based on sustained elevation of troponin-T due to findings demonstrating that early substantial elevation of troponin-T may occur without permanent myocardial injury. Electrocardiographic Q-waves have been found unreliable for diagnosis of perioperative myocardial infarction after cardiac surgery. Perioperative myocardial infarct was therefore defined by the following criteria:

Troponin-T > 2.0 μg/L on the third or fourth postoperative day

Q-wave infarct: Appearance of pathological Q-wave + biochemical markers as above or below.

In patients with recent myocardial infarct preoperatively (<1 week) or in patients with preoperatively elevated troponin-T at least one of the following were required:

Troponin-T > 2.0 μg/L on the third or fourth postoperative day and CK-MB > 50 μg/L on the first postoperative morning

Troponin-T > 2.0 μg/L on the third or fourth postoperative day and an increase of Troponin-T > 1.5 μg/L compared with preoperative levels

# **Left ventricular failure at weaning from cardiopulmonary bypass or after completion of off-pump surgery

Patients were considered to have left ventricular failure at weaning from cardiopulmonary bypass or after completion of off-pump surgery if criteria a+b or a+c or a+d were fulfilled.

a) Consensus reached by Endpoints committee that left ventricular failure was evident at weaning from cardiopulmonary bypass based on available records and hemodynamic data.

b) Cardiac index < 1,9 L/ min m^2^ BSA with SAP < 100 mm Hg

c) SvO_2_ criteria in relation to systolic arterial blood pressure (SAP) below fulfilled at weaning from cardiopulmonary bypass, five minutes after main dose of protamine sulphate and on admission to ICU that could not be explained by shivering, anemia or hypovolemia.

SvO_2_ < 50% SAP < 130 mm Hg

SvO_2_ < 55% SAP < 110 mm Hg

SvO_2_ < 60% SAP < 90 mm Hg

SvO_2_ < 65% SAP < 70 mmHg

d) Use of intraortic balloon pump or need for at least one inotropic agent in dosages listed below remaining on admission to ICU.

**SECONDARY ENDPOINTS**

Secondary endpoints covered postoperative hemodynamic state, myocardial ischemia and injury, renal function and neurological safety issues

Hemodynamic state:

Postoperative hemodynamic state, use of inotropic drugs and mechanical circulatory support, hemodynamic instability at completion of surgery^#^, incidence of severe circulatory failure^##^, need for prolonged ICU stay, cardiac mortality and late mortality.

Postoperative heart failure was categorized into heart failure evident at weaning from cardiopulmonary bypass or late circulatory failure presenting after apparently uncomplicated weaning. Late circulatory failure was classified as cardiac or non-cardiac in origin.

Myocardial ischemia and injury:

ST-depression ≥ 1 mV or ST-elevation ≥ 2 mV in the OR on EKG prior to institution of cardiopulmonary bypass, degree of myocardial injury (CK-MB postoperative day 1, troponin-T postoperative day 3 or 4).

Postoperative atrial fibrillation

Renal function:

p-Creatinine and p-Cystatin C, need for dialysis and incidence of p-creatinine elevation > 50% compared to preoperative level

Neurological safety issues:

Stroke<24 hours from surgery, stroke during hospitalization, CT-scan of the brain in suspected cases, S-100B postoperative day 3 (substudy).

**# Hemodynamic instability at completion of surgery**

Patients were considered hemodynamically unstable at completion of surgery if the attending anaesthesiologist had classified them in the institutional database as hemodynamically unstable despite use of inotropic drugs or if the needed intraaortic balloon pump. This classification was performed at the two major participating centers accounting for 788 patients out of the 861 included in the trial.

# ## Severe circulatory failure

Patients were considered to have had severe circulatory failure if criteria 1+2+3 or 2 + 4 were fulfilled.

1) ICU stay ≥ 48 hours

2) Consensus reached by Endpoints committee that heart failure presenting at weaning from cardiopulmonary bypass** or later had occurred. This was decision had to be supported by Cardiac Index or SvO_2_ data identical to those prespecified for the primary endpoint left ventricular failure at weaning from CPB.

3) Use of intraortic balloon pump or need for at least one inotropic agent in dosages listed below ≥ 24 hours after admission to ICU

4) Mortality

Dosages of inotropes required for criteria above:

Epinephrine ≥ 0,033 μg/kg BW / minute

Milrinone ≥ 0,375 μg/kg BW / minute

Dopamine ≥ 4 μg/kg BW / minute

Dobutamine ≥ 4 μg/kg BW / minute

Levosimendan regardless of dose + additional inotropic treatment in dosages above

**DATA COLLECTION**

Key study data were prospectively recorded in a CRF. Preoperatively the patients were classified with regard to CCS class and Braunwald class. Occurrence of angina at rest within 48 hours prior to surgery and occurrence of ST-segment depression ≥ 1 mm or ST-segment elevation ≥ 2 mm from admission to the OR to onset of cardiopulmonary bypass (CPB) were recorded. Troponin-T was sampled immediately before surgery and on the third postoperative day. CK-MB was sampled on the first postoperative day. Left ventricular function was assessed with transesophageal echocardiography prior to surgical incision and in association with weaning from CPB. Hemodynamic variables (mixed venous oxygen saturation, arterial saturation, heart rate, systolic and diastolic arterial pressure, diastolic pulmonary artery pressure, central venous pressure) were recorded at weaning from cardiopulmonary bypass or after termination of the last graft in off-pump surgery, five minutes after administration of protamine sulphate and on admission to ICU. Use of inotropic drugs or mechanical circulatory assist was recorded. Arterial plasma lactate was measured five minutes after administration of protamine sulphate. Plasma Cystatin-C and plasma Creatinine was sampled on the third postoperative day. The highest postoperative plasma Creatinine was also registered. CT-scan was performed in cases with suspected permanent neurological injury. Basic demographic, intraoperative and postoperative data were recorded prospectively in an institutional database (Carath) used at all participating centers.

**ROUTINES FOR UNBLINDING**

The Swedish Medical Products Agency requested meticulous surveillance and unblinding in cases of CT-verified stroke within 24 hours of surgery, mortality and suspected unexpected serious adverse reactions (SUSAR). Before unblinding was done the case was reviewed by the Clinical endpoints committee if suspected circulatory problems had occurred. Recording of adverse events was done according to Good Clinical Practice standard.

**CLINICAL MANAGEMENT**

Clinical management was standardized and similar at the three participating centers with minor differences concerning choice of anesthetic drugs. After an overnight fast patients received beta-blockers and calcium antagonists orally whereas antihypertensive and antidiabetic agents were withheld. Standard premedication consisted of orally administered flunitrazepam 0,5-1,0 mg or diazepam 5-10 mg and ketobemidone 0,1-0,2 mg/kg body weight or morphine 0.1-0.2 mg/ kg body weight. Anesthesia was induced with thiopentone (2-3 mg/ kg body weight) or propofol (2 mg/kg body weight) supplemented by a bolus dose of fentanyl 3-5 µg/kg body weight. Muscle relaxation was achieved with pancuronium 0.1 mg/kg body weight or rocuronium 0.6 mg/kg body weight. Anesthesia was maintained with isoflurane, sevoflurane or propofol supplemented with intermittent doses of fentanyl.

Standard monitoring was used consisting of 5-lead echocardiogram, pulse oximetry, continuous arterial blood pressure monitoring using a cannula in the radial artery, central venous pressure and transesophageal echocardiography. A surgical pulmonary artery catheter was introduced in all patients.

Standard surgical techniques were employed. A median sternotomy was performed in all patients. Use of cardiopulmonary bypass, single-clamp technique for aortic cross-clamping, method for myocardial protection and off-pump surgery is presented in table 1.

Postoperative sedation was achieved with propofol. Postoperative analgesia regimen consisted of ketobemidone 7-15 µg/kg body weight administered intermittent intravenously and acetaminophen 1 g every 6th hour.

Extubation was performed when body temperature reached a level above 37°C, hemodynamic values were stable including a mixed venous saturation exceeding 55 %, PO2 was above 10 kPa with FiO2 0,4 and PCO2 was below 6,5 kPa with a respiratory rate less than 30 and drainage loss was less than 100 ml per hour and declining.

**CLINICAL ENDPOINTS COMMITTEE**

The clinical endpoints committee consisted of consultants in cardiothoracic surgery and cardiothoracic anaesthesiology from each of the participating centers. The members of the committee were blinded to the treatment assignment and prespecified criteria reported in the Appendix were used to reach a consensus decision.

All cases with suspected postoperative heart failure based on SvO_2_ and other hemodynamic data, use of inotropic drugs or mechanical circulatory support, extended ICU stay, circulatory problems reported by anesthesiologists or surgeons in the clinical database were reviewed. The committee decided whether circulatory problems that met the prespecified criteria had occurred, if these circulatory problems were severe and if they were cardiac in origin, if they were evident at weaning from cardiopulmonary bypass or presented later in the postoperative course. The committee also decided if events leading to death were cardiac in origin.

**Appendix - References**

1. Dahlin LG, Kagedal B, Nylander E, Olin C, Rutberg H, Svedjeholm R: **Unspecific elevation of plasma troponin-T and CK-MB after coronary surgery**. *Scand Cardiovasc J* 2003, **37**(5):283-287.

2. Hodakowski GT, Craver JM, Jones EL, King SB, 3rd, Guyton RA: **Clinical significance of perioperative Q-wave myocardial infarction: the Emory Angioplasty versus Surgery Trial**. *J Thorac Cardiovasc Surg* 1996, **112**(6):1447-1453.

3. Svedjeholm R, Dahlin LG, Lundberg C, Szabo Z, Kagedal B, Nylander E, Olin C, Rutberg H: **Are electrocardiographic Q-wave criteria reliable for diagnosis of perioperative myocardial infarction after coronary surgery?** *Eur J Cardiothorac Surg* 1998, **13**(6):655-661.

4. Holm J, Hakanson E, Vanky F, Svedjeholm R: **Mixed venous oxygen saturation predicts short- and long-term outcome after coronary artery bypass grafting surgery: a retrospective cohort analysis**. *Br J Anaesth* 2011, **107**(3):344-350.

5. Svedjeholm R, Hakanson E, Szabo Z: **Routine SvO2 measurement after CABG surgery with a surgically introduced pulmonary artery catheter**. *Eur J Cardiothorac Surg* 1999, **16**(4):450-457.

6. Svedjeholm R, Vidlund M, Vanhanen I, Hakanson E: **A metabolic protective strategy could improve long-term survival in patients with LV-dysfunction undergoing CABG**. *Scand Cardiovasc J* 2010, **44**(1):45-58.

7. Vidlund M, Holm J, Hakanson E, Friberg O, Sunnermalm L, Vanky F, Svedjeholm R: **The S-100B substudy of the GLUTAMICS trial: Glutamate infusion not associated with sustained elevation of plasma S-100B after coronary surgery**. *Clin Nutr* 2009, **21**:358-364.
